# Supplementary material for: Detection and Genetic Characterization of Hepatitis B and D Viruses: A Multi-Site Cross-Sectional Study of People Who Use Illicit Drugs in the Amazon Region
Source: Viruses. 2021 Jul 15;13(7):1380. doi: 10.3390/v13071380 (PMC8310228; doi:10.3390/v13071380)
Supplement: Supplementary file 1 [file viruses-13-01380-s001.zip › Figure S1.pdf]

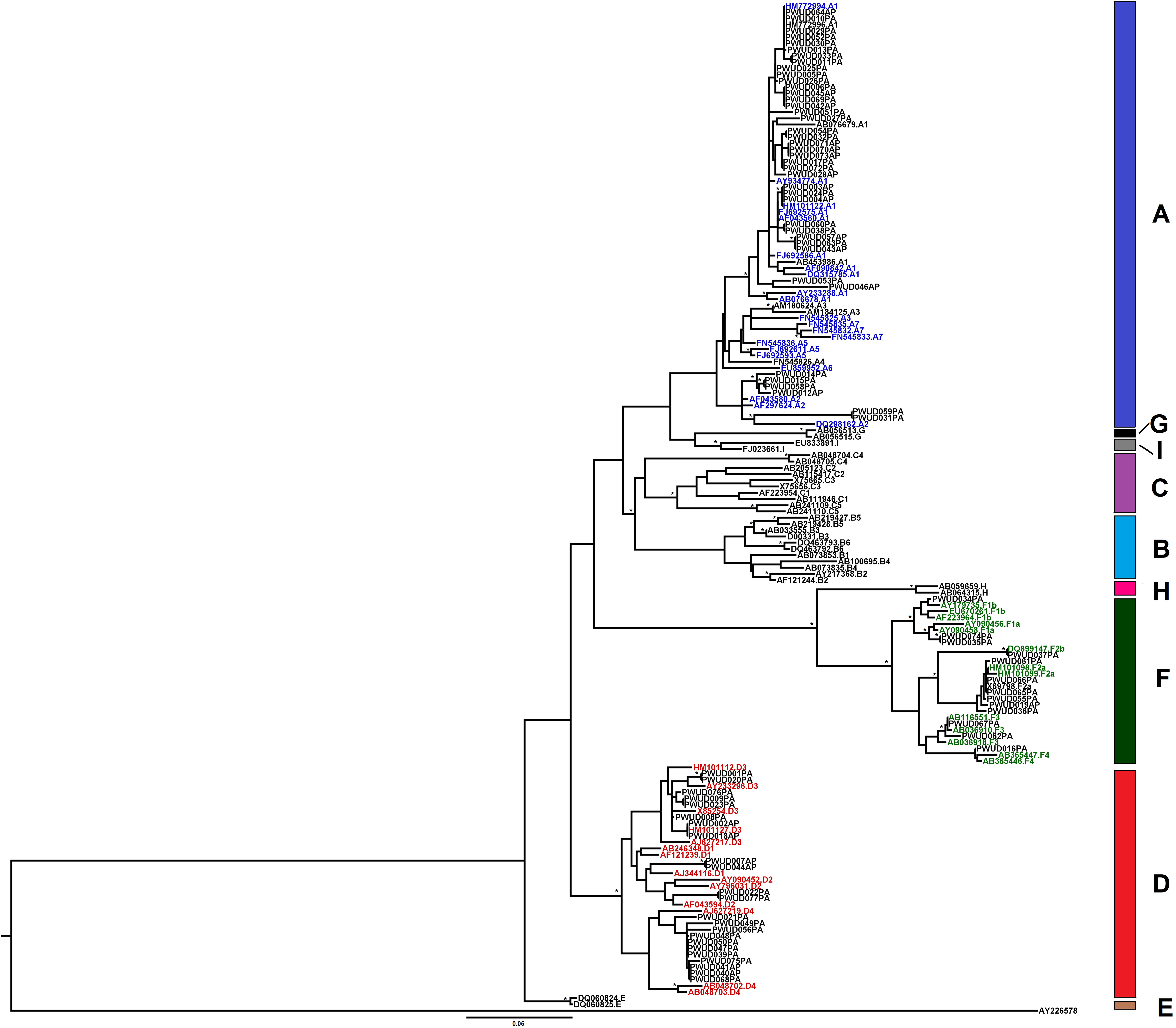

Figure S1. Maximum likelihood tree of HBV nucleotide sequences (approximately 1300 base pairs of all HBsAg and part of the polymerase genes) isolated in 77 people who use illicit drugs (PWUDs) in the Amazon region. The tree was rooted at the midpoint and contains the sequence of an Woolly monkey hepatitis B virus (AY226578), as an external group. Asterisks point to key nodes with high support (bootstrap values ≥ 0.90). Samples of this study can be identified by the acronym PWUD + number. All HBV genotypes in this phylogenetic tree were identified by different colors, and the NCBI nucleotide sequences were highlighted with their colors in the groups that contain sequences obtained in this study.
