# Supplementary material for: Detection and Genetic Characterization of Hepatitis B and D Viruses: A Multi-Site Cross-Sectional Study of People Who Use Illicit Drugs in the Amazon Region
Source: Viruses. 2021 Jul 15;13(7):1380. doi: 10.3390/v13071380 (PMC8310228; doi:10.3390/v13071380)
Supplement: Supplementary file 1 [file viruses-13-01380-s001.zip › Figure S2.pdf]

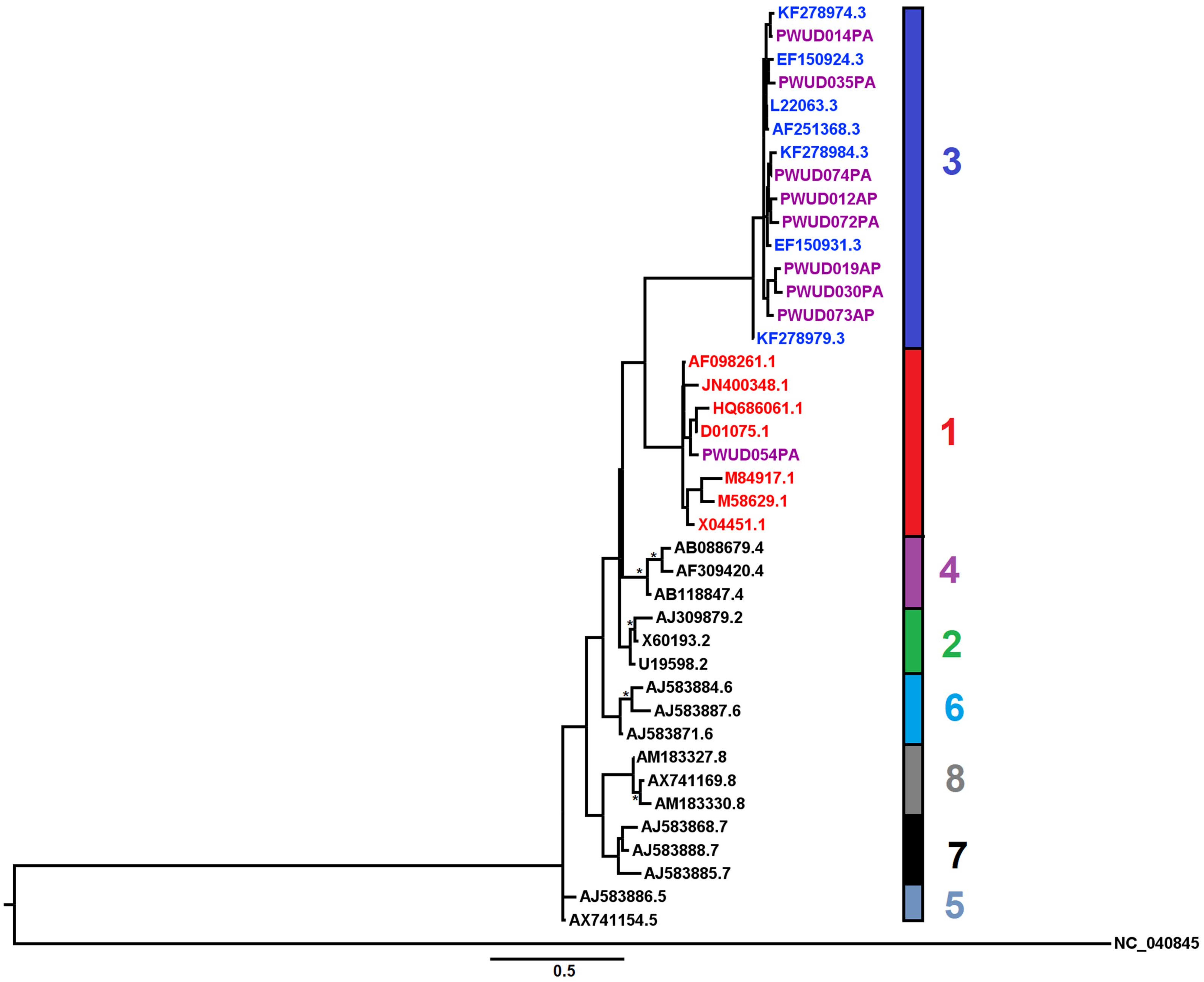

Figure S2. Phylogenetic tree of HDV nucleotide sequences (approximately 320 base pairs comprising part of the delta antigen genomic region) isolated in nine people who use illicit drugs (PWUDs) in the Amazon region. Analyses were performed by bayesian inference using the Bayesian Markov chain Monte Carlo method. The tree was rooted at the midpoint and and contains the sequence of an Avian HDV-like agent (NC040845), as an external group. Asterisks point to key nodes with high support (posterior probability values ≥ 0.90). Samples of this study can be identified by the acronym PWUD + number. All HDV genotypes in this phylogenetic tree were identified by different colors, and the NCBI nucleotide sequences were highlighted with their colors in the groups that contain sequences obtained in this study.
