# Supplementary material for: Detection and Genetic Characterization of Hepatitis B and D Viruses: A Multi-Site Cross-Sectional Study of People Who Use Illicit Drugs in the Amazon Region
Source: Viruses. 2021 Jul 15;13(7):1380. doi: 10.3390/v13071380 (PMC8310228; doi:10.3390/v13071380)
Supplement: Supplementary file 1 [file viruses-13-01380-s001.zip › Tables S1 S2.pdf]

SUPPLEMENTARY MATERIALS

Title: Detection and genetic characterization of hepatitis B and D viruses: a multi-site cross-sectional study of people who use illicit drugs in the Amazon region.

Authors: Ronylson José S. Silva, Raquel Silva do Nascimento, José Augusto J. Oliveira Neto, Fabricio Quaresma Silva, Juliana Nádia F. Piauiense, Camila Moraes Gomes, Luiz Marcelo L. Pinheiro, Rafael Lima Resque, João Renato R. Pinho, Emil Kupek, Benedikt Fischer, Luiz Fernando A. Machado, Luísa Caricio Martins, José Alexandre R. Lemos, Aldemir B. Oliveira-Filho.

Table S1. Information used for the construction of the phylogenetic trees of the hepatitis B virus (HBV) in this study.

Akaike information criterion (AIC)*	Bayesian Information Criterion (BIC)*
Model = TVM+I+G	Model = TPM3uf+G
Partition = 012314	Partition = 012012
-lnL = 14408.9885	-lnL = 4519.5605
K = 331	K = 86
FreqA = 0.2444	FreqA = 0.1630
FreqC = 0.2652	FreqC = 0.3115
FreqG = 0.2012	FreqG = 0.2650
FreqT = 0.2893	FreqT = 0.2605
R(a) [AC] = 2.3370	R(a) [AC] = 0.5556
R(b) [AG] = 4.3672	R(b) [AG] = 2.8769
R(c) [AT] = 1.2537	R(c) [AT] = 1.0000
R(d) [CG] = 0.4893	R(d) [CG] = 0.5556
R(e) [CT] = 4.3672	R(e) [CT] = 2.8769
R(f) [GT] = 1.0000	R(f) [GT] = 1.0000
p-inv = 0.3610	
Gamma shape = 0.5020	Gamma shape = 0.5330

*Information provided by the jModeltest2 software from the analysis of the alignment of the nucleotide sequences in this study and the reference sequences deposited at the NCBI.

Note: To test the robustness of the maximum likelihood tree topologies, 1,000 bootstrap replicates were performed. Bayesian analyzes consisted of two independent runs (Chain lengths of 2 x 10^6 Markov Chain Monte Carlo) with a total of 1,000 interactions and 10% burn-in.

Table S2. Information used for the construction of the phylogenetic trees of the hepatitis D virus (HDV) in this study.

Akaike information criterion (AIC)*	Bayesian Information Criterion (BIC)*
Model = TIM3+G	Model = TPM3uf+I+G
Partition = 012032	Partition = 012012
-lnL = 4518.5076	-lnL = 3809.7180
K = 87	K = 79
FreqA = 0.1709	FreqA = 0.1571
FreqC = 0.3017	FreqC = 0.3116
FreqG = 0.2741	FreqG = 0.2871
FreqT = 0.2533	FreqT = 0.2442
R(a) [AC] = 0.5553	R(a) [AC] = 0.5935
R(b) [AG] = 2.5338	R(b) [AG] = 2.8917
R(c) [AT] = 1.0000	R(c) [AT] = 1.0000
R(d) [CG] = 0.5553	R(d) [CG] = 0.5935
R(e) [CT] = 3.2090	R(e) [CT] = 2.8917
R(f) [GT] = 1.0000	R(f) [GT] = 1.0000
	P-inv = 0.3290
Gamma shape = 0.5340	Gamma shape = 1.7530

*Information provided by the jModeltest2 software from the analysis of the alignment of the nucleotide sequences in this study and the reference sequences deposited at the NCBI.

Note: To test the robustness of the maximum likelihood tree topologies, 1,000 bootstrap replicates were performed. Bayesian analyzes consisted of two independent runs (Chain lengths of 2 x 10^6 Markov Chain Monte Carlo) with a total of 1,000 interactions and 10% burn-in.
